# circRNAs in drug resistance of breast cancer

**DOI:** 10.32604/or.2022.027547

**Published:** 2023-01-31

**Authors:** SEMA MISIR, SERAP OZER YAMAN, NINA PETROVIĆ, CEREN SUMER, CEYLAN HEPOKUR, YUKSEL ALIYAZICIOGLU

**Affiliations:** 1 Department of Biochemistry, Faculty of Pharmacy, Sivas Cumhuriyet University, Sivas, 58010, Turkey; 2 Department of Medical Biochemistry, Karadeniz Technical University Faculty of Medicine, Trabzon, 61000, Turkey; 3 Laboratory for Radiobiology and Molecular genetics, “VINČA” Institute of Nuclear Sciences-National Institute of the Republic of Serbia, University of Belgrade, Belgrade, 11000, Serbia; 4 Department of Experimental Oncology, Institute for Oncology and Radiology of Serbia, Belgrade, 11000, Serbia; 5 Comprehensive Cancer Center, Faculty of Life Science & Medicine, King’s College London, London, SE11UL, United Kingdom

**Keywords:** Breast cancer, Chemotherapy, circRNAs, Drug resistance, Non-coding RNA

## Abstract

Breast cancer (BC) is the most common heterogeneous disease in women and one of the leading causes of cancer-related death. Surgery, chemotherapy, radiotherapy, hormone, and targeted therapy are the gold standards for BC treatment. One of the significant challenges during the treatment of BC represents resistance to chemotherapeutics, resistance that severely limits the use and effectiveness of the drugs used for BC treatment. Therefore, it is essential to develop new strategies to improve therapeutic efficacy. Circular RNAs (circRNAs) are a large group of non-coding RNAs that covalently form closed circular loops by joining their 5′, and 3′; ends. Accumulating evidence suggests that circRNAs have a vital role in cancer development, progression, and BC resistance to chemotherapy. The purpose of this review is to discuss the biological properties of circRNAs, and how circRNAs induce resistance to conventional therapeutic anti-cancer drugs used in BC treatment, by emphasizing and summarizing the potential roles of circRNAs in mechanisms of drug resistance, such as drug efflux, apoptosis dysfunction, autophagy, and DNA damage repair. CircRNAs are associated with drug resistance via ATP-binding cassette (ABC) efflux transporters, while some others by inhibition of cell apoptosis, thus leading to resistance to tamoxifen in BC cells. In contrast, others are involved in the promotion of BC cells chemoresistance by doxorubicin-induced autophagy. CircRNAs may have clinical significance in regulating or overcoming BC drug resistance and may give directions towards a novel approach to personalized BC treatment. CircRNAs may significantly contribute to the identification of new therapeutic targets for the prevention of BC chemoresistance.

## Introduction

Breast cancer (BC) is still one of the most common female malignancies worldwide [1]. Surgery followed by chemo and, or radiotherapy, endocrine (hormone) therapy, targeted therapy, and immunotherapy is the gold standard for BC treatment. Tumor, node, metastasis (TNM) stage, tumor grade, the status of receptors for estrogen and progesterone (ER and PR), respectively, and human epidermal growth factor receptor 2 (Her-2) are used to select an adequate therapeutic modality. Subclassification based on intrinsic molecular subtypes, recognizes Luminal A, B, or Her-2-enriched (hormone receptor-positive or negative), Her-2 positive, and triple-negative breast cancer (TNBC) [2,3]. Patients with Her-2 positive status are predominantly treated with trastuzumab and frequently with another anti- Her-2 agent-lapatinib in combination with chemotherapy. TNBCs are considered a particular BC subtype with poor response to therapy and higher chances of developing resistance to systemic chemotherapy and radiation treatment [4]. A metastatic disease that leads to organ failure is a significant cause of cancer-related deaths [5]. Patients with TNBC, predominantly basal-like, mainly benefit from neoadjuvant anthracycline/taxane chemotherapy (up to one-third of patients respond to treatment) [3].

Clinical studies have also shown that basal-like and non-basal-like TNBC patients exert various responses to multi-agent, combined chemotherapy in different stages [6]. For example, carboplatin or bevacizumab in combination with paclitaxel (PAX) and then with doxorubicin (DOX) and cyclophosphamide may significantly improve the response to the treatment of TNBC in stages II and III [7]. Besides all treatment modalities, many tumors are resistant to the treatment or develop resistance over time [8]. Resistance to therapy can be intrinsic or acquired during the treatment. Patients with metastasis and advanced clinical stages are more likely to develop resistance to standard chemotherapeutics-taxanes and anthracyclines, and tyrosine kinase inhibitors. Patients with ER and, or progesterone receptor-positive tumors are usually treated with adjuvant endocrine therapy, predominantly with tamoxifen (TAM), which has a limited duration of its effectiveness, approximately up to 5 years. After that period, the disease recurrence rates and mortality increase, due to the development of resistance to treatment [9]. In addition, drug resistance can occur by different mechanisms such as multi-drug resistance (MDR), inhibition of cell death (apoptosis suppression), alterations in drug metabolism, epigenetics and drug targets, DNA repair, and enhancement of gene amplification. Important mechanisms leading to drug resistance are: (i) changes in the function and content of pharmacological drug targets; (ii) increased drug efflux due to the over-increased expression of plasma membrane transporters; (iii) modified drugs; (iv) enhanced DNA repair and, accordingly, accelerated cellular repair mechanisms; (v) functional changes in mechanisms of cell death; and (vi) alterations/changes in cell proliferation and cell cycle [10,11].

Advances in DNA and RNA microarray and sequencing technologies, and the development of targeted and personalized therapies, as well as advances in proteomics technology provide new strategies to overcome drug resistance and contribute to this field [12]. In recent studies, circular RNAs (circRNAs) have been shown to play essential roles in BC development and pathogenesis. Expression levels of circRNAs vary in different cancer types, and altered expression of circRNAs play a vital role in tumor initiation and progression [13]. The role of circRNAs in proliferation, migration, invasion and especially chemoresistance of cancer cells is shown to be displayed through the regulation of the expression of related miRNAs and targeted genes [12,14]. For example, hsa_circ_0000199 is associated with response to chemotherapy by inhibiting phosphatidylinositol-3-kinase/Akt and the mammalian target of rapamycin (PI3K/AKT/mTOR) pathway through miR-613 and miR-206. Still, it was shown on triple-negative BC [15]. In the basis of resistance to BC therapy might stand genetic and epigenetic changes, including circular RNA level changes.

The purpose of this review is to discuss the biological properties of circRNAs and BC drug resistance mechanisms associated with circRNAs. In addition, the limitations of the knowledge held and its potential future aspects are discussed. We believe that circRNAs will contribute to the identifying of new potential therapeutic targets for the prevention and treatment of chemoresistance.

## CircRNA Biogenesis and Functions

**Figure 1:**
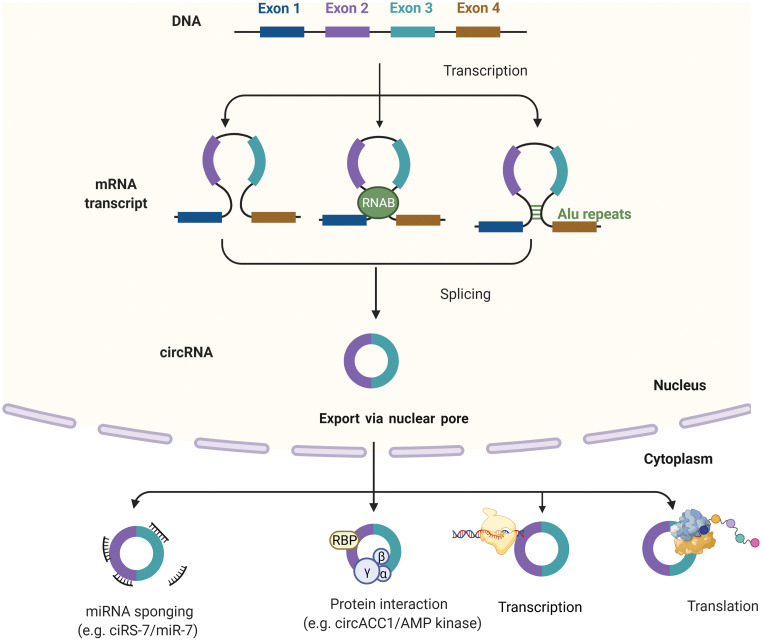
Biogenesis and function of circRNAs. Biogenesis of circRNAs; Intron pairing model, Intron pairing-driven circularization and RBP-induced cyclization. CircRNAs can be classified into four types circRNA, EciRNA, EIciRNA, ciRNAs. circRNAs can act as miRNA sponges, transcriptional regulators, binding partners of proteins, or even translated into functional proteins. Created with BioRender

Circular RNA derives from eukaryotic protein-coding genes as a form of loop-RNA, formed by joining 5′ and 3′ ends covalently to form a single-strand circular structure [16]. CircRNA can contain exons, introns or both in their structure and are formed by back-splicing mechanisms. Exonic circRNA forms when flanking regions of the two introns containing exons are positioned between them close to the circular structure with the help of ribonucleoproteins (Fig. 1). Exonic-intronic circRNAs form from sequences containing several introns and exons. When complementary sequences of introns bring up two exons near each other, they join at the splice sites, discarding ending introns and retaining the intron between them. Pure intronic circRNA originates from Alu repeats joining, which when inverted, are complementary with each other [17].

Most circRNAs are localized in the cytoplasm, and a small fraction of them can be found in the nucleus. Their localization depends on the cricRNA sequence length. After the maturation, circRNAs exit from the nucleus through the complex of the nuclear pore [18]. Some intronic nuclear cricRNA can supervise the regulation of genes they originated from (parental genes) [19]. CircRNAs are involved in and regulate transcription, splicing, and translation of paternal genes (Fig. 1). CircRNA affects the process of translation by terminating or pausing transcription, by recruiting some splicing factors or by interacting with ribonucleoproteins and with RNA Pol II, thus enhancing promoter activity and parental gene expression [20]. The second *modus operandi* of circRNA is similar to miRNA sponging and competing endogenous RNA (ceRNAs) activities. They can catch both miRNAs and mRNAs, as well. CircRNAs cannot be described as entirely non-coding RNA because, under some circumstances, they can be translated into proteins, the ones with internal ribosome entry sites (IRES), enabling them to recruit ribosomes. But, it should be denoted that proteins translated from circRNAs, are mostly non-functional because they represent the shorted versions of original proteins translated from paternal genes [16]. By their ability to interact with proteome in so many combinations in the physicochemical and biological manner (interaction with chromatin and various enzymes), they can be described as essential regulators of cancer formation, progression, and future biomarkers of response to therapy, or maybe in the future as targets for therapy.

## Mechanisms of Drug Resistance

**Figure 2:**
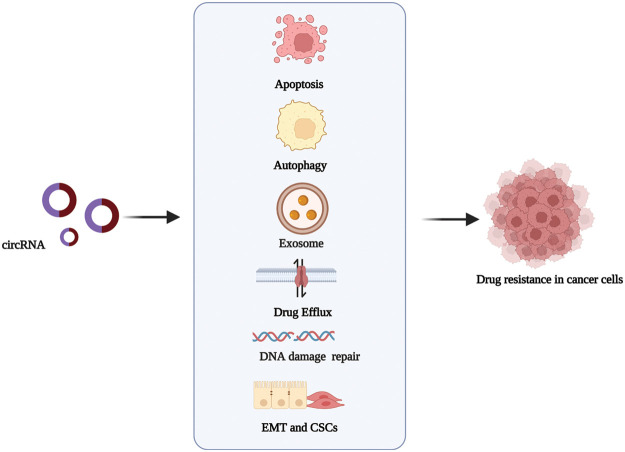
The mechanisms via which circRNAs regulate drug resistance in cancer. circRNAs promote or inhibit the resistance to antitumor drugs in different types of cancer by influencing cell apoptosis, drug efflux, exosome, autophagy, DNA repair, and epithelial-mesenchymal transition. Created with BioRender

Anti-cancer drug resistance is a complex process resulting from altering drug targets. Drug resistance can occur before or as a result of cancer treatment [21]. In recent years, critical advances have been made in the research and development of targeted and personalized therapy drugs, as well as chemotherapeutic drugs with multiple factors, such as immune response inhibitors. However, drug resistance mechanisms still seriously hinder the role, functioning, and effectiveness of these drugs in cancer treatment [10]. A holistic understanding of the functions of circRNAs in the molecular mechanisms that lead to drug resistance will help develop better strategies for cancer therapy. The most widely described mechanisms in circRNA-related drug resistance are shown in Fig. 2.

## Drug Efflux

Efflux pump mechanisms ensure that critical physiological functions such as preventing toxin absorption in the gastrointestinal tract, elimination of bile, functioning of the blood-brain and placental barrier, and renal excretion of drugs work effectively [22]. Drug efflux from cancer cells is associated with drug or multi-drug resistance mechanisms. One of the barriers to the success of cancer chemotherapies remains drug resistance [23]. The adenosine triphosphate (ATP) binding cassette (ABC) is a class of transmembrane transporters. These carriers, P-glycoproteins (P-GP), include breast cancer resistance protein (BCRP) and multi-drug resistance protein 1 (MRP1) [24]. Membrane-localized pumps, including proteins P-GP/MDR1, MRP1, MRP2, and BCRP, have functional roles in the drug efflux mechanism [25]. In cancerous tissues, the expression level of the MDR1 gene encoded by P-GP is upregulated by increasing. Moreover, when overexpression mechanisms of MDR1 were examined, it was revealed that DOX treatment could trigger a significant increase in MDR1 expression levels in lung cancer cells without affecting expression in normal respiratory cells [26]. Lung, prostate gland, and mammary gland tissues do not express MDR1. Drug resistance in these tissues is associated explicitly with BCRP and MRP1 and is also controlled by other members of the ABC transporter family [25,27]. As a result, these three carriers are both related to each other and protect cancer cells from various first-line chemotherapies was revealed. ABC transporters are frequently overexpressed in cancer cells and significantly limit the effective delivery of chemotherapy. They form a defense system against various cytotoxic agents and chemotherapeutic drugs. In this mechanism, ABC transporters can actively transport intracellular drugs across the membrane in an inverse concentration gradient, pump chemotherapeutic drugs from tumor cells, and reduce intracellular drug accumulation [28]. ATP-binding cassette superfamily G member 2 (ABCG2), BCRP, is responsible for regulating and altering the intracellular distribution of drugs. Mediates drug resistance through glutathione-dependent drug efflux so drugs cannot reach their targets [29].

The function of some circRNAs in drug resistance of cancers associated with ABC efflux transporters was discovered [14]. In osteosarcoma, the knockdown of circ-CHI3L1.2 down-regulates the expression levels of P-GP, MRP1 and glutathione S-transferase P1 (GSTP1) and has been found to weaken cisplatin (CDDP) resistance [30]. ATP binding cassette subfamily B member 1 (ABCB1) overexpression appeared to reverse the effects of circRNA_103615 silencing on CDDP resistance [31]. Therefore, it is crucial to explore the relationships between circRNAs, ABC efflux transporters, and tolerance to anti-cancer drugs to find new therapeutic targets for cancer.

## Inhibition of Cell Apoptosis

Apoptosis represents an evolutionarily conserved, highly complex, central mechanism of cell death, an intrinsic death program that is important in maintaining tissue homeostasis in development and adulthood, and is also a tumor suppressor mechanism [32]. Cancer chemotherapy aims to induce apoptosis of cancer cells. In this way, it can make them targets for anti-cancer drugs [33]. The primary cell death mechanism activated by chemotherapy drugs is apoptosis. It triggers apoptosis through two main pathways, the intrinsic mitochondrial and the extrinsic transmembrane pathway. The effector phase of apoptosis includes several pro-apoptotic proteins (e.g., Bcl-2-associated X protein (BAX), Bcl-2 homologous antagonist killer (BAK), BH3 interacting-domain death agonist (BID), Bcl-2 Interacting mediator of cell death (BIM), BCL2 associated agonist of cell death (BAD), and p53 upregulated modulator of apoptosis (PUMA)) and anti-apoptotic proteins (e.g., B-cell lymphoma-2 (BCL-2), B-cell lymphoma extra-large (BCL-XL), and Induced myeloid leukemia cell differentiation protein (MCL-1)) as intrinsic pathways. This pathway is under the control of the BCL-2 family. The extrinsic pathway is regulated by the tumor necrosis factor (TNF) receptor family [34,35]. A potential mechanism of drug resistance mediates the activation of these anti-apoptotic pathways, resulting in the suppression of apoptotic signal transduction. Thus, the mechanism of drug resistance is caused by the emergence of resistance to chemotherapeutics [14]. CircRNAs, which have a highly stable thermodynamic structure, act on and modulate pro- or anti-apoptotic proteins has been stated. They regulate the apoptosis of drug-resistant cancer cells via these pathways emphasized [36]. For example, circAMOTL1 was seen to regulate the expression of AKT-related pro-apoptotic BAX and BAK and anti-apoptotic BCL-2 proteins in BC. It has thus been shown to modulate the PAX resistance, particularly in BC [37]. circRNA_0006528 has an effective potency in tamoxifen-resistant cells in BC. Knockdown of this circRNA has been found to reduce the IC50 of paclitaxel-resistant cells. circRNA_0006528 was found to reduce proliferation, migration, and autophagy and induce apoptosis in these cells [38]. Also, as a result of gain and loss analysis, the knockdown of circ-ABCB10 and DUSP7 activates let-7a was found. This activation appeared to increase the paclitaxel sensitivity of BC cells. Thus, it showed that apoptosis could increase and have an inhibitory effect due to the excessive increase in PAX sensitivity [39]. Thus, determining the influential roles of circRNAs that modulate pro- or anti-apoptotic proteins to promote apoptosis of cancer cells and reduce tolerance to anti-cancer drugs is a critical research topic.

## Autophagy

Autophagy is a cellular process that maintains cell homeostasis by recycling damaged or useless proteins or organelles through a lysosome-dependent degradation system in normal cells. This ensures an advantage for cancer cells in tumorigenesis. Autophagy could play a dual role by promoting cancer cell survival as well as autophagic cancer cell death in the development of cancer cells [40]. Although drug resistance mechanisms induced by autophagy have not been fully understood, it has been indicated that enhanced autophagy plays a crucial role in the resistance to chemo-radiotherapy and targeted therapy in various carcinoma cells, primarily via its cytoprotective function through multiple mechanisms mediated by autophagy-related genes, MDR, heat shock proteins, survival-related signaling pathways, miRNAs [41–43]. Emerging evidence indicated that circular RNAs also regulate autophagy, which could promote drug resistance in various tumors. For instance, combining imatinib with autophagy inhibition reduces acquired resistance in gastrointestinal stromal tumors [44]. The cirEIF6 and hsa_circ_0092276 promote chemoresistance by modulating autophagy induced by cisplatin and doxorubicin in thyroid and breast cancer cells, respectively [45,46].

## CircRNA Associated with DNA Damage Repair

DNA damage response and repair (DDRR) is a strategy of the cell to maintain the integrity of the genome after the impact of various genotoxic events. To reduce genome instability and repair double or single-strand breaks (DSB or SSB), the cell activates molecules from repair signaling cascade pathways [47]. DSB induces Homologous Recombination (HR) and Non-Homologous End Joining (NHEJ). Irregular DSB repair leads to chromatin remodeling and can cause genome instability, which is one of the mechanisms underlying the basis of malignant transformation [48]. Several circRNAs are shown to be associated with DNA damage response and regulation of DNA repair. It has been shown that some circRNAs, such as circSMARCA5 interact with DNA exons. circSMARCA5 is derived from the gene coding for SWI/SNF Related, Matrix Associated, Actin Dependent Regulator of Chromatin, Subfamily A, Member 5 (SMARCA5) protein which contributes to the structural maintenance of the chromatin structure near the DNA damage, thus enabling components of DNA repair machinery to access the damaged regions. Moreover, higher circSMARCA5 levels were detected in cancer cell lines compared with standard BC cells and in BC samples compared with normal tissue [49]. circMET RNA overexpression was associated with malignant transformation. In MCF-7 and T47D cells, circMET was significantly higher in tamoxifen-resistant cells compared with cells sensitive to TAM. Also, treatment with tamoxifen decreased the cell viability sensitive MCF-7 and T47D, but without effect on resistant cell lines. Furthermore, circMET was associated with resistance to tamoxifen via targeting miR-204-5p targeting, which increases aryl hydrocarbon receptor (AHR), an inhibitor of DNA-double strand break repair leading to increased cell viability and decreased sensitivity to TAM [50].

## EMT and CSCs

Epithelial-mesenchymal transition (EMT) is characterized by the loss of epithelial phenotype and the acquisition of mesenchymal features [51]. EMT is well known to facilitate malignant progression by promoting migratory and invasive behavior in multiple cancer types [52]. In addition, emerging evidence suggests that EMT appears as a significant contributor to drug resistance to multiple therapeutic agents in many different preclinical models [53]. Such resistance has been linked to EMT-mediators, which are specific transcription factors (Zinc finger protein SNAI1 (Snail), Twist-related protein 1 (Twist), Zinc Finger E-Box Binding Homeobox 1 (Zeb1)), posttranscriptional regulators (miRNAs), or posttranslational regulators (such as Casein kinase I (CK1)) in a wide variety of cancer types [54–59] and attributed to different mechanisms including elevated expression of ABC transporters, evading apoptosis signaling pathways, and decreased cell proliferation [60]. For instance, the downregulation of EMT-inducing transcription factors such as Twist, Snail, and Forkhead Box C2 (FOXC2) makes invasive breast cancer cells more chemosensitive by reducing their expression of ABC transporters [61].

Epithelial-mesenchymal transition is closely associated with acquiring the cancer stem cell (CSC) phenotype in various types of cancer, including breast, lung, pancreatic, and colon carcinomas [60,62–64]. Therefore, CSCs-associated drug resistance mechanisms are very similar to those related to EMT [60]. For instance, the overexpression of Twist in breast cancer cells has been shown to increase the formation of a breast cancer stem cell phenotype and enhance the expression of ABCC1 transporters [65]. EMT might cause drug resistance by promoting CSCs characteristics. It has been shown that the spontaneous conversion of HER2+ breast carcinoma cells to a cluster of differentiation 44, CD44+ HER2-basal/mesenchymal phenotype via EMT could lead to trastuzumab resistance [66]. While conventional therapeutics successfully eradicate non-CSCs, CSC-enriched carcinoma cells can remain viable even in the presence of therapeutic reagents and promote tumor recurrence and resistance to drugs.

In recent years, it has been shown that circular RNAs have been associated with EMT and CSCs in different types of cancers [67–70]. Although the relationship between circRNAs and EMT in drug resistance remains to be elucidated, there is an increasing number of reported data that circRNAs are involved in drug resistance by promoting EMT and stemness. For instance, the Hepatocyte growth factor (HGF)/c-mesenchymal-epithelial transition factor (c-Met), HGF/c-Met signaling pathway regulates the cirRNA CCDC66 expression to promote EMT and cisplatin resistance in lung adenocarcinoma cells [71]. It has been reported that circUBE2D2 enhances EMT progression and tamoxifen resistance in tamoxifen-resistant BC cells [72]. circ-PVT1 promotes paclitaxel resistance of gastric cancer by increasing the expression of ZEB1, an EMT-inducing regulator, through miR-124-3p [73].

Although it is thus clear that circRNAs help cancer cells escape from therapeutics by regulating EMT and stemness, more data is required to study the drug resistance induced by circRNAs. Therefore, EMT-inducing regulators, including circRNAs, may emerge as helpful therapeutic strategies to tackle drug resistance.

## CircRNA from Exosomal Cargoes

Exosomes belong to a class of extracellular vesicles between 30–100 nm in size. Exosomes are membrane-derived packages containing various molecules, including lipids, proteins, and nucleic acids in their cargo [74]. The most important role of exosomes is to carry information and mediate communication between the parental and recipient cell, thus enabling inter or intracellular information and molecule transfer in endocrine, paracrine and autocrine manner [75]. Exosomes form via double invagination of the cell membrane and pack into multicellular vesicular bodies (MVB) by endocytosis. Then, proteins named endosomal sorting complexes required for transport (ESCRT) direct cargo sorting of exosomes, microvesicles, apoptotic bodies, and other particles into MVBs and directly release outside the parental cell by exocytosis [76,77].

Loading cargo into exosomes is probably cell-tissue specific and depends on proteins involved in the packing process [78]. Exosomes can contain a significant proportion of various RNA molecules, which is also cell-type specific [79]. Messenger RNA, miRNA, and other small and long non-coding RNAs, including circRNA can be found in exosomes. RNAs found in exosomes can modulate the activity of recipient cells, thus influencing intracellular processes and signaling pathways. Sequencing of RNA sheds light not only on circRNA presence in exosomes, but also on their origin, and parental cells, and revealed the presence of thousands of circRNAs in exosomes extracted from human sera [80]. Exosomes originating from tumor cells can travel to distant organs, thus having the potential to induce metastasis formation or progression [74]. So, exosomal trafficking and transport might be an additional mechanism influencing cancer formation and regulation of the disease progression. In exosomes, circRNA can sponge and bind to miRNA molecules, thus changing their downstream silencing of mRNAs and their expression and activity, as well as in the cytoplasm.

For example, circCDR1-AS exerts its sponging function in exosomes via sponging miR-7 [80], which can, in turn, increase the malignant and metastatic potential of BC. miR-7 was higher in metastatic breast cancer than in localized and BC compared with standard control samples [81]. It has been shown by Hu et al. that exosome-released hsa_circ_UBE2D2 was overrepresented in tamoxifen-resistant MCF-7 BC cells *in vitro* and *in vivo*. They have also shown that hsa_circ_UBE2D2 exerted its role in the increase of resistance to tamoxifen by targeting miR-200a-3p [72], indicating that it could be one of the targets for overcoming tamoxifen resistance which frequently occurs over time, after the treatment of ER-positive BCs.

## CircRNAs and Drug Resistance

**Figure 3:**
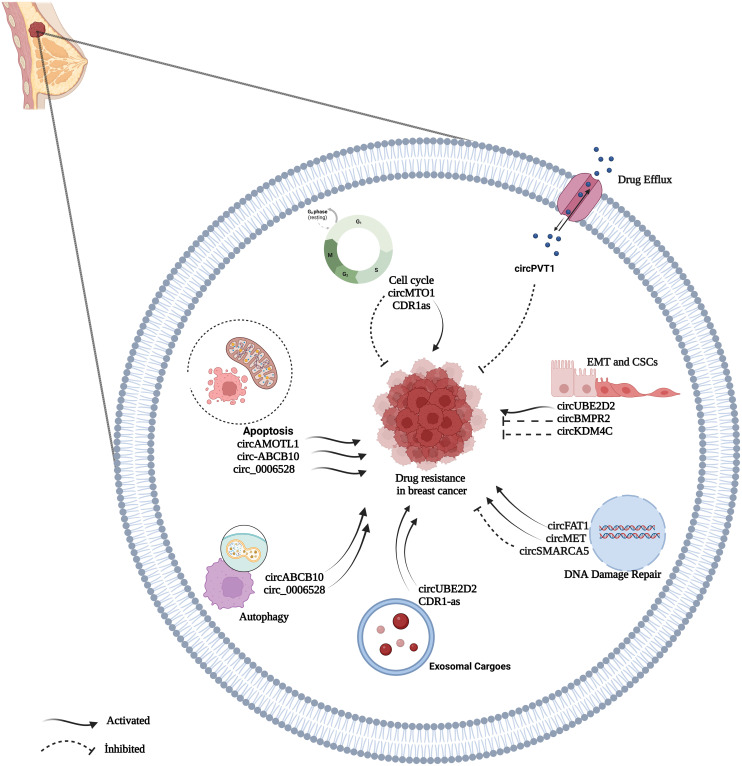
A summary diagram of some circRNAs involved in the drug resistance of BC. circRNAs could participate in drug resistance of BC by drug efflux, apoptosis dysfunction, cell cycle, exosome, autophagy, DNA damage repair, and epithelial-mesenchymal transition. Created with BioRender

As BC is a very heterogeneous disease with high mortality and morbidity [82], multidisciplinary approaches to treatment such as surgery, chemotherapy, radiation therapy, hormone therapy, and targeted therapy are used as treatment gold standard [83]. Chemotherapy, in particular, is an effective method for treating patients with breast cancer as an adjuvant or neoadjuvant therapeutic approach [17,84]. Chemotherapy drugs include PAX, TAM, CDDP, DOX, and 5-fluorouracil (5-FU), and there are also specific chemotherapeutic, endocrine, and targeted drugs used frequently in BC patients [82,85]. Despite the significant advances in therapeutics, the intended improvement cannot be achieved at the desired level [17]. One of the most important reasons for that is drug resistance, which restricts the efficacy of chemotherapy [82,85]. In BC, drug resistance is regulated by various mechanisms [85]. Identifying the key molecules in these processes may play an essential role in understanding the formation of resistance and reversing the resistance developed [86]. Many recent studies revealed that circRNAs play regulating role in drug resistance in various cancer [84,87]. Understanding the functions of circRNAs in resistance mechanisms will help identify new potential therapeutic targets for drug-resistant tumors and enable new approaches for tumor treatment [84,88]. The circRNAs related to drug resistance in BC are shown in Table 1. Some of the most recognized circRNA involved in drug resistance in BC are shown in Fig. 3.

**Table 1:** CircRNAs related to drug resistance in BC

circRNAs	Cirbase ID	Expression	Target/pathway	Drug	Ref
circUBAP2	–	Up	miR-300/ASF1B/PI3K/AKT/mTOR	Cisplatin	[89]
CDR1as	Hsa_circ_0001946	Up	miR-7/PSME3	Cisplatin	[90]
circSMARCA5	Hsa_circ_0001445	Down	–	Cisplatin	[49]
circCDK1	–	Up	miR-489-3p/CDK1	Tamoxifen	[91]
–	Hsa_circ_0025202	Down	miR-182-5p/FOXO3a	Tamoxifen	[92]
–	Hsa_circ_0025202	Down	miR-197-3p/HIPK3	Tamoxifen	[93]
–	Hsa_circ_0097922	Up	miR-876-3p/ACTN4	Tamoxifen	[94]
circUBE2D2	–	Up	miR-200a-3p	Tamoxifen	[72]
circBMPR2	Hsa_circ_0003218	Down	miR-553/USP4	Tamoxifen	[95]
circAMOTL1	–	Up	AKT1	Paclitaxel	[37]
circRNF111	Hsa_circ_0001982	Up	miR-140-5p/E2F3	Paclitaxel	[96]
circGFRA1	–	Up	miR-361-5p/TLR4	Paclitaxel	[97]
circABCB10	–	Up	Let-7a-5p/DUSP7	Paclitaxel	[39]
–	Hsa_circ_0006528	Up	miR-1299/CDK8	Paclitaxel	[98]
circHIPK3	Hsa_circ_0000284	Up	miR-1286/HK2	Paclitaxel	[99]
circWAC	–	Up	miR-142/WWP1	Paclitaxel	[100]
CDR1as	Hsa_circ-0001946	Up	miR-7/CCNE1	5-fluorouracil (5‑FU)	[101]
circFBXL5	–	Up	miR‑216b/HMGA2	5-fluorouracil (5‑FU)	[102]
circMMP11	–	Up	miR-153-3p/ANLN	Lapatinib	[103]
circFAT1	–	Up	miRNA-525-5p/SKA1	Oxaliplatin	[104]
circMTO1	Hsa_circ_007874	Down	TRAF4/Eg5	Monastrol	[105]
circBGN.	–	Up	SLC7A11	Trastuzumab	[106]
circCDYL2	–	Up	GRB7/FAK	Trastuzumab	[107]
–	Hsa_circ_0001598	Up	miR-1184/PD-L1	Trastuzumab	[108]
circABCB1 circEPHA3.1 circEPHA3.2	-	Up Down Down	PI3K/Akt, AGE-RAGE	Docetaxel	[109]
circKDM4C		Down	miR-548p/PBLD	Doxorubicin	[110]
circATXN7	Hsa_circ_0066436	Up	miR-149-5p/HOXA11	Doxorubicin	[111]
–	Hsa_circ_0044556	Up	miR‑145/NRAS	Doxorubicin	[112]
circUBE2D2	Hsa_circ_0005728	Up	miR‑512‑3p/CDCA3	Doxorubicin	[113]
–	Hsa_circ_0092276	Up	miR-348/ATG7	Doxorubicin	[46]
–	Hsa_circ_0001667	Up	miR-4458/NCOA3	Doxorubicin	[114]
–	Hsa_circ_0006528	Up	miR-7-5p/Raf1	Doxorubicin	[115]

## Paclitaxel

Paclitaxel, a Taxol derivative, is one of the chemotherapy drugs used commonly for the treatment of breast cancer [37]. PAX is effective against early and metastatic BC and acts through the stabilization of the microtubules and inhibition of the cell division [99]. Although PAX is an effective drug, resistance to PAX is inevitable in most patients at the terminal phase after a 6 to 10-month treatment [37]. PAX resistance becomes a significant clinical problem in treating patients with breast cancer. Therefore, it is vital to understand the molecular mechanisms of PAX resistance to develop therapeutic interventions [97]. circAMOTL1 was stated to be potentially responsible for PAX resistance in breast cancer cells. circAMOTL1 was shown to suppress cell apoptosis through anti- and pro-apoptotic protein expressions by regulating the AKT pathway, suggesting that circAMOTL1 may be a new potential target of breast cancer treatment to reduce the resistance to PAX [37]. A study conducted by Zang et al. showed that circ-RNF111 increased significantly in PAX-resistant BC tissues and cells, and the silencing of circ-RNF111 resulted in the inhibition of cell viability, colony formation, and invasion, thereby the blockage of cell growth and the increased PAX sensitivity. The circ-RNF111/miR-140-5p/E2F3 (E2F Transcription Factor 3) axis was suggested to be a new promising regulation network for PAX resistance [96]. circGFRA1, highly expressed in PAX-resistant cells, was shown to act as miRNA sponge to inhibit the miR-361-5p expression. circGFRA1 was indicated to have a regulating effect on miR-361-5p/TLR4 and thus affect the sensitivity of TNBC cells against PAX [97]. In another study, it was noted that circ-ABCB10 contributed to the PAX resistance of BC cells through Let-7a-5p/DUSP7 axis. It was noted that it increased PAX sensitivity and apoptosis while inhibiting the invasion and autophagy of BC cells following the down-regulation of circ-ABCB10 [39]. The other studies in the literature stated that BC cells became sensitive to PAX treatment through the regulation of the miR-1299/CDK8 (Cyclin Dependent Kinase 8) axis as a result of the downregulation of circ_0006528 [98], and the regulation of miR-1286/HK2 axis as a consequence of the downregulation of circHIPK3 [99]. In addition, the down-regulation of circWAC was stated to increase the sensitivity of TNBC cells to PAX. It was suggested that circWAC/miR-142/WWP1 (WW Domain Containing E3 Ubiquitin Protein Ligase 1) axis might be a new biomarker and therapeutic target for TNBC [100].

## Tamoxifen

The estrogen receptor is positive in approximately 70% of breast cancers [116]. TAM is a hormone antagonist used as an (ER) positive first-line adjuvant endocrine therapy in BC patients and has been used for treating patients for nearly thirty years [92]. TAM functions by inhibiting the estrogen binding to ERs and blocking the stimulation signals modulated by ERs [91]. Acquired resistance develops in nearly 40% of the patients receiving TAM [116]. The mechanisms of TAM resistance are regulated by many factors. It was stated that these factors include non-coding RNAs, including circRNAs, and that these molecules may be potential targets for developing new therapeutic approaches [93]. circCDK1 expression was shown to increase in Tamoxifen-resistant breast cancer tissues and cells. The silencing of circCDK1 attenuated tamoxifen-resistant and inhibited the proliferation and survival of the resistant cells. Moreover, circCDK1 was also indicated to be involved in the TAM resistance mechanism through the circCDK1/miR-489-3p/CDK1 regulating network by targeting miR-489-3p [91]. Hsa_circ_0025202 is expressed in BC tissues at low levels. Hsa_circ_0025202 reduced the resistance to TAM by acting as a miR-182-5p sponge to regulate the activity and expression of the Forkhead transcription factor of subfamily member 3a (FOXO3a), the target gene [92]. Another study demonstrated that hsa_circ_0025202 suppressed carcinogenesis in BC by regulating the miR-197-3p/HIPK3 (Homeodomain-interacting protein kinase 3) axis and may increase the sensitivity to TAM [90]. Furthermore, hsa_circ_0097922 is overexpressed in TAM-resistant BC cells. Hsa_circ_0097922 has been suggested to inhibit tumor development in BC by interacting with miR-876-3p and may increase the sensitivity to TAM [94]. Silencing of circBMPR2 increased tamoxifen resistance in BC cells. It has been reported to inhibit the proliferation, migration, and invasion of BC cells via the circBMPR2/miR-553/USP4 (Ubiquitin specific protease 4) axis [95]. In another study, circ_UBE2D2 was isolated from exosomes bound to miR-200a-3p, thereby increasing the TAM resistance of BC cells [72].

## Cisplatin

Cisplatin is a chemotherapeutic drug used for the treatment of a wide variety of cancers, including lung, bladder, ovarian, liver, and breast cancer [117,118]. Although cisplatin exerts considerable therapeutic effect, the resistance develops after long-term use and thus causes cancer recurrence and decreased overall survival. Demonstrating the effect of circRNAs on cisplatin resistance in recent years and understanding the underlying mechanisms will provide potential new strategies to improve the therapeutic efficiency in breast cancer treatment [117]. circUBAP2 was shown to enhance the expression of cisplatin-resistant TNBC, and the TNBC sensitivity against cisplatin may be increased by silencing the circUBAP2. Moreover, circUBAP2 was indicated to regulate the CDDP sensitivity of TNBC through the miR-300/ASF1B (Anti-silencing function 1) axis. circUBAP2 also targeted miR-300 to regulate ASF1B expression, thereby decreasing protein levels of ASF1B significantly. It was noted that ASF1B activated PI3K/AKT/mTOR signaling to facilitate CDDP resistance of TNBC cells. These findings showed the functional role of circUBAP2/miR-300/ASF1B/PI3K/AKT/mTOR (PAM) pathway in CDDP-resistant TNBC [89]. In another study, the overexpression of Cerebellar degeneration-related protein 1 (CDR1) was reported to be associated with adverse chemotherapeutic effects, and the sensitivity of the drug-resistant breast cancer cells to cisplatin was increased through the involvement of competitive inhibition of miR-7 and REGγ [90]. Xu et al. [49] reported the effect of circSMARCA5 (hsa_circ_0001445) on the sensitivity of BC to cisplatin. circSMARCA5 was present in the tissue and plasma samples of BC patients at a lower level than normal samples, and the restoration of circSMARCA5 levels enhanced the chemosensitivity of BC cells [49].

## Doxorubicin (Adriamycin)

Doxorubicin is one of the chemotherapeutic drugs used commonly for the treatment of BC. However, the development of chemoresistance restricts its clinical use. It is generally used alone or concomitantly with other drugs, such as docetaxel, for the chemotherapy of BC. The reasons for the resistance of BC to chemotherapy are closely related to the recurrence and metastasis of breast cancer [119].

Wang et al. [111] reported that circATXN7/miR-149-5p/homeobox A11 (HOXA11) axis plays a role in the development of breast cancer and doxorubicin resistance. circATXN7 increased the activation of HOXA11 by targeting miR-149-5p, accelerated the formation of the tumor, and supported the doxorubicin resistance [111]. circKDM4C down-regulated in doxorubicin-resistant breast cancer cells. The overexpression of circKDM4C resulted in the inhibition of proliferation, migration, and invasion of the cancer cells and the improvement of doxorubicin sensitivity. The therapeutic targeting of circKDM4C/miR-548p/PBLD axis may be a promising treatment approach for BC patients [110]. circRNA _0044556 was highly expressed in TNBC cells and especially DOX-resistant cells. circRNA _0044556 supported cell viability, DOX-resistance, and migration while sponging miR-145 and inhibiting apoptosis due to its overexpression. The down-regulation of circ_0044556 increased the DOX-susceptibility of TNBC cells, thereby leading to decreased cell viability, increased apoptosis, and reduced migration [112]. In another study, circUBE2D2 plays an oncogenic role in TNBC, which it supports by sponging miR-512–3p thus preventing targeting Cell division cycle associated 3 (*CDCA3*) expression by sponging miR-512–3p- and that it induces which leads to doxorubicin resistance in TNBC. The down-regulation of circUBE2D2 decreased DOX-resistance by regulating miR-512-3p/CDCA3 axis. circUBE2D2 silencing may be a novel therapeutic approach for TNBC treatment [113]. Hsa_circ_0092276 was overexpressed in DOX-resistant BC cells. Overexpression of hsa_circ_0092276 inhibited the effect of DOX on the proliferation and apoptosis of BC cells. Hsa_circ_0092276 supported autophagy and DOX resistance in breast cancer by regulating the miR 348/ATG7 (Autophagy related 7) axis [46]. Cui et al. [114] indicated that circ_0001667 was overexpressed in DOX-resistant BC tissues and cells. After silencing circ_0001667, the malignant progression and chemotherapeutic resistance of DOX-resistant breast cancer were inhibited by the miR-4458/NCOA3 (Nuclear receptor coactivator 3) axis [114]. circ_0006528 was overexpressed in DOX-resistant tissues and cells, and also increased the DOX sensitivity of BC cells due to silencing the circ_0006528. circ_0006528 targets miR-7-5p that leads to overexpression of Raf1 [115]. Raf1 plays a role in the drug resistance of BC cells by regulating the ERK pathway [120].

## 5-fluorouracil

5-fluorouracil is commonly used for the treatment of many cancer types, including breast cancer [121,122]. 5-FU acts on nucleoside metabolism; 5-FU is incorporated into RNA and DNA, leading to cytotoxicity and cell death [121]. However, this resistance restricts the efficacy of the drug. It is essential, to identify the mechanisms in regulating the pathogenesis of 5-FU resistance against BC and chemoresistance. circRNAs also play a role in the regulation of chemoresistance, to 5-FU. Zhu et al. showed that the role of circFBXL5 in 5-FU resistance of BC, and circFBXL5 was overexpressed in resistant BC cells. circFBXL5 supported 5-FU resistance of breast cancer by regulating the miR-216b/HMGA2 (High mobility group AT-Hook 2) axis [102]. Another study showed that Cerebellar degeneration related 1 (*CDR1*) mRNA as a target of miR-7 and 5-FU affects chemosensitivity in BC cells. CDR1 regulates the chemosensitivity of BC cells by inhibiting cyclin E1 (*CCNE*) [101].

## Other Drugs

Lapatinib is a tyrosine kinase inhibitor [103] and is an agent used in combination with capecitabine or taxanes for metastatic breast cancer [123]. Albeit its low side effects, lapatinib resistance limits the efficiency of treatment. Wu et al. showed that the role of circ-MMP11, emerged as an oncogene, in lapatinib resistance in breast cancer. circ-MMP11 was carried via exosomes and may increase the lapatinib resistance by regulating miR-153-3p/ANLN anillin network in BC [103].

Oxaliplatin (OX) is a platin analog and shows its mechanism of action by disrupting DNA synthesis [104]. OX is used commonly for the clinical treatment of BC [104], and unlike other platin compounds, it does not have nephrotoxicity and minimal myelosuppression [124]. circFAT1 level increased in OX-resistant BC tissues and cells, and that circFAT1/miR-525-5p/SKA1 (Spindle and kinetochore-associated protein 1) axis increased the OX resistance in BC via activation of Neurogenic locus notch homolog protein (Notch) and Wingless/Integrated (Wnt) pathway [104].

Docetaxel (DTX) is a semi-synthetic paclitaxel derivative and is regarded as a second-generation taxane [125]. Its mechanism of action takes place via the binding of guanosine diphosphate, GDP-bound tubulin [126], and thus cell cycle arrest and formation of apoptosis [127]. Taxane-containing treatment was shown to improve general survival both in an the early phase and terminal phases of breast cancer [128]. However, drug resistance, which may be resulted from mutations in drug-resistance genes or post-transcription mechanisms, may develop in many patients receiving taxanes. Huang et al. reported that circRNAs could sponge chemotherapy-associated miRNAs and arrange signaling pathways contributing to docetaxel resistance in BC cells. Eight chemotherapy-associated miRNAs can be sponged by circABCB1, circEPHA3.1, and circEPHA3.2. These three circRNAs contribute to docetaxel resistance via the PI3K-Akt and Advanced glycation end-products/Receptor for advanced glycation end-products (AGE-RAGE) signaling pathways [109].

Monastrol is a molecule that stops the cells in mitosis by specifically inhibiting Eg5, a member of the Kinesin-5 family [129]. The drugs targeting mitotic spindles are among the most effective cancer therapeutics. circRNA‑MTO1 was down-regulated in Monastrol-resistant cells. The overexpression of circRNA‑MTO1 suppressed cell viability, supported monastrol-based cell cytotoxicity, and increased the sensitivity to monastrol [105].

Trastuzumab (TRA) is a targeted monoclonal antibody treatment preferred in patients with HER2-positive BC. However, it has been observed that there are cases that do not show sensitivity to HER2 treatment or that go from susceptible to drug-resistant. This situation broadly inhibits the efficiency of TRA. Therefore, understanding the mechanism underlying drug resistance is vital. The literature review revealed that circ-BGN was associated with poor prognosis in TRA-resistant BC tissues. circ-BGN was overexpressed in TRA-resistant BC tissue, and its down-regulation decreased cell viability and especially restoration of the sensitivity to TRA [106]. In another study, circCDYL2, which is overexpressed in TRA-resistant patients and provides *in vitro* and *in vivo* TRA resistance in BC cells, was identified. circCDYL2 plays a role in the formation of the GRB7-FAK complex. The circCDYL2-GRB7-FAK complex plays a critical role in maintaining the HER2 signal and contributes to TRA resistance [107]. Li et al. reported that hsa_circ_0001598 is an oncogenic circRNA that plays a vital role in regulating PD-L1 expression and results in the immune escape and resistance of TRA in BC [108].

## CircRNA-Based Therapy Strategies

According to the evidence we presented above, circRNAs play prominent roles in BC resistance to chemotherapy. Their high stability, abundance, and diversity render circRNAs promising biomarkers [130]. With tissue/cell type specificity, aberrant expression, and the association with cancer development and drug resistance in a wide variety of cancers, circRNAs have emerged as possible therapeutic targets over time for overcoming the drug resistance we described above [14,131]. Due to the differential dysregulation of circRNAs in cancer, recent studies have focused on both overexpression and knockdown strategies to target circRNAs. RNA interference-based strategies using siRNA, shRNA, and altered antisense oligonucleotides targeting back-spliced junction (BSJ) of upregulated circRNAs associated with drug resistance could be one approach [132]. The targeting of such circRNAs might enhance the sensitivity of breast cancer patients to chemotherapeutic drugs. Accumulating evidence revealed that BC chemoresistance (DOX, PAX, TAM) are reversed by applying siRNA or shRNA targeting oncogenic circRNAs by serving as miRNA sponges in BC cells or patient-derived xenograft mouse models [14,37,97,113]. Recently, there have been few studies showing another efficient approach in depleting circRNAs specifically and robustly would be the cre-lox system [133], and clustered regularly interspaced short palindromic repeats/CRISPR-associated protein 13 (CRISPR/Cas13)-mediated knockout without affecting their linear counterparts [134] in retinal pericytes and hepatocellular carcinoma, respectively. He et al. [135] have published a detailed report regarding these systems. These strategies could be translated to breast cancer cells or patient-derived animal models.

Conversely, circRNA expression vectors, including plasmids, lentiviral and adenoviral vectors have been used to enhance the concentration of downregulated circRNAs in cultured cells and mice. For instance, the ectopic expression of Foxo3 circular RNA has been shown to suppress tumor growth, cell proliferation, and survival with the sponging effect on miRNAs in breast carcinoma [136]. Further, Adeno-associated viruses were exploited to express circITCH to reduce cardiotoxicity by sponging miR-330-5p in DOX-treated mice [137]. Another simple and effective strategy to reintroduce circRNAs would be directly synthesizing artificial circRNAs of interest that can be delivered directly into target cells to sponge miRNAs [138].

Finally, to improve therapeutic efficiency, nanoparticle and exosome-based delivery systems could be exploited to increase the delivery of circRNAs, circRNA expression vectors, or circRNA-targeting agents, especially in reducing the adverse effects arising from off-target tissues and preventing immune activation in breast cancer [135]. For instance, Du et al. have used an AuNPs-conjugated siRNA targeting circDnmt1, resulting in the inhibition of cellular autophagy and tumor growth in breast cancer [139]. Moreover, the ever first-ever lipid nanoparticle-siRNA drug, named patisiran, has been approved for the treatment of hereditary transthyretin amyloidosis [140]. On the other hand, a study proposed that oxaliplatin-resistant colorectal cancer cells released ciRS-122 enriched in their exosomes into sensitive cells, resulting in drug resistance via miR-122 sponging. In this study, si-ciRS-122 delivered by an exosome-based system was shown to reverse this drug resistance by modulating the ciRS-122-miR-122-PKM2 (Pyruvate kinase isozyme M2) axis *in vivo* [141]. Thus, these systems might be exploited as promising tools in reversing breast cancer resistance.

## Conclusion and Perspectives

Chemotherapy is an essential method for the clinical treatment of particular BC subtypes, but the development of drug resistance limits the effectiveness of the treatment and causes its failure. Therefore, it is crucial to elucidate the molecular pathways underlying resistance to reduce or eliminate drug resistance. In recent years, many studies have indicated that circRNAs play essential roles in tumorigenesis, development, and drug resistance. In this review, recent studies on the effects of circRNAs on BC and drug resistance are summarized. Many circRNAs were revealed to function as miRNA sponges and regulate the function of the circRNA-miRNAs-mRNA axis or participate in drug resistance mechanisms by stimulating oncogenic signaling pathways. This field of study is limited to *in vitro* and *in vivo* experiments. However, the mechanisms are still unclear, and with the new resistance mechanisms to be determined, the importance of circRNAs in the field will increase. Additional circRNAs need to be discovered and associated with drug resistance in BC, and most of the circRNAs reported in BC have been only related to a couple of well-known drugs. Moreover, there is no such study on comprehensive expression patterns of circRNAs in resistant drug-resistant and sensitive BC patients. There are also significant limitations, including off-target gene knockdown, side effects of Cas13 expression, and toxicity of nanoparticles. As these limitations are addressed with new studies before the clinical use of circRNA-based therapeutic strategies in BC, circRNAs will be much more promising as effective therapeutic targets in the field. Further studies will provide important information about the understanding of molecular mechanisms and their clinical utility. We believe that the knowledge of the effects of circRNAs on drug resistance will also provide new approaches to the treatment of BC.

## References

[1] Sung, H., Ferlay, J., Siegel, R. L., Laversanne, M., Soerjomataram, I. et al. (2021). Global cancer statistics 2020: GLOBOCAN estimates of incidence and mortality worldwide for 36 cancers in 185 countries. CA: A Cancer Journal for Clinicians*,* 71 *(*3*),* 209–249. DOI 10.3322/caac.21660. 33538338

[2] Biswal, A., Erler, J., Qari, O., Topilow, A. A., Gupta, V. et al. (2019). The effect of the new eighth edition breast cancer staging system on 100 consecutive patients. Journal of Clinical Medicine Research*,* 11 *(*6*),* 407–414. DOI 10.14740/jocmr3803. 31143307 PMC6522240

[3] Prat, A., Pineda, E., Adamo, B., Galván, P., Fernández, A. et al. (2015). Clinical implications of the intrinsic molecular subtypes of breast cancer. Breast*,* 24 *(*2*),* 26–35. DOI 10.1016/j.breast.2015.07.008. 26253814

[4] Wu, Q., Siddharth, S., Sharma, D. (2021). Triple negative breast cancer: A mountain yet to be scaled despite the triumphs. Cancers*,* 13 *(*15*),* 3697. DOI 10.3390/cancers13153697. 34359598 PMC8345029

[5] Riggio, A. I., Varley, K. E., Welm, A. L. (2021). The lingering mysteries of metastatic recurrence in breast cancer. British Journal of Cancer*,* 124 *(*1*),* 13–26. DOI 10.1038/s41416-020-01161-4. 33239679 PMC7782773

[6] Sikov, W. M., Berry, D. A., Perou, C. M., Singh, B., Cirrincione, C. T. et al. (2015). Impact of the addition of carboplatin and/or bevacizumab to neoadjuvant once-per-week paclitaxel followed by dose-dense doxorubicin and cyclophosphamide on pathologic complete response rates in stage II to III triple-negative breast cancer: CALGB 40603 (Alliance). American Journal of Clinical Oncology*,* 33 *(*1*),* 13–21. DOI 10.1200/JCO.2014.57.0572. PMC4268249 25092775

[7] Shepherd, J. H., Ballman, K., Polley, M. Y. C., Campbell, J. D., Fan, C. et al. (2022). CALGB 40603 (Alliance): Long-term outcomes and genomic correlates of response and survival after neoadjuvant chemotherapy with or without carboplatin and bevacizumab in triple-negative breast cancer. American Journal of Clinical Oncology*,* 40 *(*12*),* 1323–1334. DOI 10.1200/JCO.21.01506. PMC9015203 35044810

[8] Luque-Bolivar, A., Pérez-Mora, E., Villegas, V. E., Rondón-Lagos, M. (2020). Resistance and overcoming resistance in breast cancer. Breast Cancer*,* 12*,* 211–229. DOI 10.2147/BCTT.S270799. 33204149 PMC7666993

[9] Yang, L. H., Tseng, H. S., Lin, C., Chen, L. S., Chen, S. T. et al. (2012). Survival benefit of tamoxifen in estrogen receptor-negative and progesterone receptor-positive low grade breast cancer patients. Journal of Breast Cancer*,* 15 *(*3*),* 288–295. DOI 10.4048/jbc.2012.15.3.288. 23091541 PMC3468782

[10] Cree, I. A., Charlton, P. (2017). Molecular chess? Hallmarks of anti-cancer drug resistance. BMC Cancer*,* 17 *(*1*),* 1–8. DOI 10.1186/s12885-016-2999-1. 28056859 PMC5214767

[11] Reygaert, C. W. (2018). An overview of the antimicrobial resistance mechanisms of bacteria. AIMS Microbiology*,* 4 *(*3*),* 482–501. DOI 10.3934/microbiol.2018.3.482. 31294229 PMC6604941

[12] Pavlíková, L., Šereš, M., Breier, A., Sulová, Z. (2022). The roles of microRNAs in cancer multidrug resistance. Cancers*,* 14 *(*4*),* 1090. DOI 10.3390/cancers14041090. 35205839 PMC8870231

[13] Zhang, M., Xin, Y. (2018). Circular RNAs: A new frontier for cancer diagnosis and therapy. Journal of Hematology and Oncology*,* 11 *(*1*),* 21. DOI 10.1186/s13045-018-0569-5. 29433541 PMC5809913

[14] Liu, X. Y., Zhang, Q., Guo, J., Zhang, P., Liu, H. et al. (2022). The role of circular RNAs in the drug resistance of cancers. Frontiers in Oncology*,* 11*,* 1–14. DOI 10.3389/fonc.2021.790589. PMC8766647 35070998

[15] Li, H., Xu, W., Xia, Z., Liu, W., Pan, G. et al. (2021). Hsa_circ_0000199 facilitates chemo-tolerance of triple-negative breast cancer by interfering with miR-206/613-led PI3K/Akt/mTOR signaling. Aging*,* 13 *(*3*),* 4522–4551. DOI 10.18632/aging.202415. 33495420 PMC7906206

[16] Zhou, W. Y., Cai, Z. R., Liu, J., Wang, D. S., Ju, H. Q. et al. (2020). Circular RNA: Metabolism, functions and interactions with proteins. Molecular Cancer*,* 19 *(*1*),* 1–19. DOI 10.1186/s12943-020-01286-3. 33317550 PMC7734744

[17] He, X., Xu, T., Hu, W., Tan, Y., Wang, D. et al. (2021). Circular RNAs: Their role in the pathogenesis and orchestration of breast cancer. Frontiers in Cell and Developmental Biology*,* 9*,* 647736. DOI 10.3389/fcell.2021.647736. 33777954 PMC7991790

[18] Huang, C., Liang, D., Tatomer, D. C., Wilusz, J. E. (2018). A length-dependent evolutionarily conserved pathway controls nuclear export of circular RNAs. Genes & Development*,* 32 *(*9–10*),* 639–644. DOI 10.1101/gad.314856.118. 29773557 PMC6004072

[19] Li, Z., Huang, C., Bao, C., Chen, L., Lin, M. et al. (2015). Exon-intron circular RNAs regulate transcription in the nucleus. Nature Structural and Molecular Biology*,* 22 *(*3*),* 256–264. DOI 10.1038/nsmb.2959. 25664725

[20] Geng, X., Jia, Y., Zhang, Y., Shi, L., Li, Q. et al. (2020). Circular RNA: Biogenesis, degradation, functions and potential roles in mediating resistance to anticarcinogens. Epigenomics*,* 12 *(*3*),* 267–283. DOI 10.2217/epi-2019-0295. 31808351

[21] Zahreddine, H., Borden, K. L. B. (2013). Mechanisms and insights into drug resistance in cancer. Frontiers in Pharmacology*,* 4*,* 1–8. DOI 10.3389/fphar.2013.00028. 23504227 PMC3596793

[22] Ughachukwu, P., Unekwe, P. (2012). Efflux pump-mediated resistance in chemotherapy. Annals of Military and Health Sciences Research*,* 2 *(*2*),* 191. DOI 10.4103/2141-9248.105671. PMC3573517 23439914

[23] Gong, J., Jaiswal, R., Mathys, J. M., Combes, V., Grau, G. E. R. et al. (2012). Microparticles and their emerging role in cancer multidrug resistance. Cancer Treatment Reviews*,* 38 *(*3*),* 226–234. DOI 10.1016/j.ctrv.2011.06.005. 21757296

[24] Chen, Z., Shi, T., Zhang, L., Zhu, P., Deng, M. et al. (2016). Mammalian drug efflux transporters of the ATP binding cassette (ABC) family in multidrug resistance: A review of the past decade. Cancer Letters*,* 370 *(*1*),* 153–164. DOI 10.1016/j.canlet.2015.10.010. 26499806

[25] Emran, T. B., Shahriar, A., Mahmud, A. R., Rahman, T., Abir, M. H. et al. (2022). Multidrug resistance in cancer: Understanding molecular mechanisms, immunoprevention, and therapeutic approaches. Frontiers in Oncology*,* 12*,* 891652. DOI 10.3389/fonc.2022.891652. 35814435 PMC9262248

[26] Pesic, M., Markovic, J. Z., Jankovic, D., Kanazir, S., Markovic, I. D. et al. (2006). Induced resistance in the human non small cell lung carcinoma (NCI-H460) cell line in vitro by anticancer drugs. Journal of Chemotherapy, 18 (1), 66–73. DOI 10.1179/joc.2006.18.1.66. 16572896

[27] Haber, M., Smith, J., Bordow, S. B., Flemming, C., Cohn, S. L. et al. (2006). Association of high-level MRP1 expression with poor clinical outcome in a large prospective study of primary neuroblastoma. American Journal of Clinical Oncology*,* 24 *(*10*),* 1546–1553. DOI 10.1200/JCO.2005.01.6196. 16575006

[28] Gillet, J. P., Efferth, T., Remacle, J. (2007). Chemotherapy-induced resistance by ATP-binding cassette transporter genes. Biochimica et Biophysica Acta-Reviews on Cancer*,* 1775 *(*2*),* 237–262. DOI 10.1016/j.bbcan.2007.05.002. 17572300

[29] Domenichini, A., Adamska, A., Falasca, M. (2019). ABC transporters as cancer drivers: Potential functions in cancer development. Biochimica et Biophysica Acta-General Subjects*,* 1863 *(*1*),* 52–60. DOI 10.1016/j.bbagen.2018.09.019. 30268729

[30] Zhang, Z., Zhou, Q., Luo, F., Zhou, R., Xu, J. et al. (2021). Circular RNA circ-CHI3L1.2 modulates cisplatin resistance of osteosarcoma cells via the miR-340-5p/LPAATβ axis. Human Cell*,* 34 *(*5*),* 1558–1568. DOI 10.1007/s13577-021-00564-6. 34164774

[31] Liang, H., Lin, Z., Lin, H., Zhao, L., Huang, W. (2021). circRNA_103615 contributes to tumor progression and cisplatin resistance in NSCLC by regulating ABCB1. Experimental and Therapeutic Medicine*,* 22 *(*3*),* 1–8. DOI 10.3892/etm.2021.10366. PMC8281253 34306203

[32] Morana, O., Wood, W., Gregory, C. D. (2022). The apoptosis paradox in cancer. International Journal of Molecular Sciences*,* 23 *(*3*),* 1328. DOI 10.3390/ijms23031328. 35163253 PMC8836235

[33] Lomonosova, E., Chinnadurai, G. (2009). BH3-only proteins in apoptosis and beyond: An overview. Oncogene*,* 27 *(*S1*),* 2–19. DOI 10.1038/onc.2009.39. PMC2928556 19641503

[34] Pommier, Y., Sordet, O., Antony, S., Hayward, R. L., Kohn, K. W. (2004). Apoptosis defects and chemotherapy resistance: Molecular interaction maps and networks. Oncogene*,* 23 *(*16*),* 2934–2949. DOI 10.1038/sj.onc.1207515. 15077155

[35] An, X., Sarmiento, C., Tan, T., Zhu, H. (2017). Regulation of multidrug resistance by microRNAs in anti-cancer therapy. Acta Pharmaceutica Sinica B*,* 7 *(*1*),* 38–51. DOI 10.1016/j.apsb.2016.09.002. 28119807 PMC5237711

[36] Ghazimoradi, M. H., Babashah, S. (2022). The role of CircRNA/miRNA/mRNA axis in breast cancer drug resistance. Frontiers in Oncology*,* 12*,* 966083. DOI 10.3389/fonc.2022.966083. 36132137 PMC9484461

[37] Ma, J., Fang, L., Yang, Q., Hibberd, S., Du, W. W. et al. (2019). Posttranscriptional regulation of AKT by circular RNA angiomotin-like 1 mediates chemoresistance against paclitaxel in breast cancer cells. Aging*,* 11 *(*23*),* 11369–11381. DOI 10.18632/aging.102535. 31819016 PMC6932896

[38] Hao, J., Du, X., Lv, F., Shi, Q. (2021). Knockdown of circ_0006528 suppresses cell proliferation, migration, invasion, and adriamycin chemoresistance via regulating the miR-1236-3p/CHD4 axis in breast cancer. Journal of Surgical Research*,* 260 *(*204*),* 104–115. DOI 10.1016/j.jss.2020.10.031. 33333383

[39] Yang, W., Gong, P., Yang, Y., Yang, C., Yang, B. et al. (2020). Circ-ABCB10 contributes to paclitaxel resistance in breast cancer through Let-7a-5p/DUSP7 axis. Cancer Management and Research*,* 12*,* 2327–2337. DOI 10.2147/CMAR.S238513. 32273769 PMC7108723

[40] Shin, D. W. (2020). Dual roles of autophagy and their potential drugs for improving cancer therapeutics. Biomolecules & Therapeutics*,* 28 *(*6*),* 503–511. DOI 10.4062/biomolther.2020.155. 33077698 PMC7585634

[41] Yoon, J. H., Ahn, S. G., Lee, B. H., Jung, S. H., Oh, S. H. (2012). Role of autophagy in chemoresistance: Regulation of the ATM-mediated DNA-damage signaling pathway through activation of DNA-PKcs and PARP-1. Biochemical Pharmacology*,* 83 *(*6*),* 747–757. DOI 10.1016/j.bcp.2011.12.029. 22226932

[42] Pagotto, A., Pilotto, G., Mazzoldi, E. L., Nicoletto, M. O., Frezzini, S. et al. (2017). Autophagy inhibition reduces chemoresistance and tumorigenic potential of human ovarian cancer stem cells. Cell Death and Disease*,* 8 *(*7*),* e2943. DOI 10.1038/cddis.2017.327. 28726781 PMC5550872

[43] Xu, J. L., Yuan, L., Tang, Y. C., Xu, Z. Y., Xu, H. D. et al. (2020). The role of autophagy in gastric cancer chemoresistance: Friend or foe? Frontiers in Cell and Developmental Biology*,* 8*,* 621428. DOI 10.3389/fcell.2020.621428. 33344463 PMC7744622

[44] Gupta, A., Roy, S., Lazar, A. J. F., Wang, W. L., McAuliffe, J. C. et al. (2010). Autophagy inhibition and antimalarials promote cell death in gastrointestinal stromal tumor (GIST). Proceedings of the National Academy of Sciences of the United States of America*,* 107 *(*32*),* 14333–14338. DOI 10.1073/pnas.1000248107. 20660757 PMC2922542

[45] Liu, F., Zhang, J., Qin, L., Yang, Z., Xiong, J. et al. (2018). Circular RNA EIF6 (Hsa_circ_0060060) sponges miR-144-3p to promote the cisplatin-resistance of human thyroid carcinoma cells by autophagy regulation. Aging*,* 10 *(*12*),* 3806–3820. DOI 10.18632/aging.101674. 30540564 PMC6326687

[46] Wang, Q., Liang, D., Shen, P., Yu, Y., Yan, Y. et al. (2021). Hsa_circ_0092276 promotes doxorubicin resistance in breast cancer cells by regulating autophagy via miR-348/ATG7 axis. Translational Oncology*,* 14 *(*8*),* 101045. DOI 10.1016/j.tranon.2021.101045. 34023560 PMC8163983

[47] Papaspyropoulos, A., Hazapis, O., Lagopati, N., Polyzou, A., Papanastasiou, A. D. et al. (2021). The role of circular rnas in dna damage response and repair. Cancers*,* 13 *(*21*),* 5352. DOI 10.3390/cancers13215352. 34771517 PMC8582540

[48] Gorgoulis, V. G., Pefani, D. E., Pateras, I. S., Trougakos, I. P. (2018). Integrating the DNA damage and protein stress responses during cancer development and treatment. Journal of Pathology*,* 246 *(*1*),* 12–40. DOI 10.1002/path.5097. 29756349 PMC6120562

[49] Xu, X., Zhang, J., Tian, Y., Gao, Y., Dong, X. et al. (2020). CircRNA inhibits DNA damage repair by interacting with host gene. Molecular Cancer*,* 19 *(*1*),* 1–19. DOI 10.1186/s12943-020-01246-x. 32838810 PMC7446195

[50] Huang, X. Y., Zhang, P. F., Wei, C. Y., Peng, R., Lu, J. C. et al. (2020). Circular RNA circMET drives immunosuppression and anti-PD1 therapy resistance in hepatocellular carcinoma via the miR-30-5p/snail/DPP4 axis. Molecular Cancer*,* 19 *(*1*),* 1–18. DOI 10.1186/s12943-020-01213-6. 32430013 PMC7236145

[51] Dongre, A., Weinberg, R. A. (2019). New insights into the mechanisms of epithelial-mesenchymal transition and implications for cancer. Nature Reviews Molecular Cell Biology*,* 20 *(*2*),* 69–84. DOI 10.1038/s41580-018-0080-4. 30459476

[52] Nieto, M. A., Huang, R. Y. Y. J., Jackson, R. A. A., Thiery, J. P. P. (2016). EMT: 2016. Cell*,* 166 *(*1*),* 21–45. DOI 10.1016/j.cell.2016.06.028. 27368099

[53] de Las Rivas, J., Brozovic, A., Izraely, S., Casas-Pais, A., Witz, I. P. et al. (2021). Cancer drug resistance induced by EMT: Novel therapeutic strategies. Archives of Toxicology*,* 95 *(*7*),* 2279–2297. DOI 10.1007/s00204-021-03063-7. 34003341 PMC8241801

[54] Park, S. M., Gaur, A. B., Lengyel, E., Peter, M. E. (2008). The miR-200 family determines the epithelial phenotype of cancer cells by targeting the E-cadherin repressors ZEB1 and ZEB2. Genes & Development*,* 22 *(*7*),* 894–907. DOI 10.1101/gad.1640608. 18381893 PMC2279201

[55] Arumugam, T., Ramachandran, V., Fournier, K. F., Wang, H., Marquis, L. et al. (2009). Epithelial to mesenchymal transition contributes to drug resistance in pancreatic cancer. Cancer Research*,* 69 *(*14*),* 5820–5828. DOI 10.1158/0008-5472.CAN-08-2819. 19584296 PMC4378690

[56] Xu, Y., Lee, S. H., Kim, H. S., Kim, N. H., Piao, S. et al. (2010). Role of CK1 in GSK3Β-mediated phosphorylation and degradation of Snail. Oncogene*,* 29 *(*21*),* 3124–3133. DOI 10.1038/onc.2010.77. 20305697

[57] Haslehurst, A. M., Koti, M., Dharsee, M., Nuin, P., Evans, K. et al. (2012). EMT transcription factors snail and slug directly contribute to cisplatin resistance in ovarian cancer. BMC Cancer*,* 12 *(*1*),* 91. DOI 10.1186/1471-2407-12-91. 22429801 PMC3342883

[58] Fischer, K. R., Durrans, A., Lee, S., Sheng, J., Li, F. et al. (2016). Epithelial-to-mesenchymal transition is not required for lung metastasis but contributes to chemoresistance. Nature*,* 527 *(*7579*),* 472–476. DOI 10.1038/nature15748. PMC4662610 26560033

[59] Kang, E., Seo, J., Yoon, H., Cho, S. (2021). The post-translational regulation of epithelial-mesenchymal transition-inducing transcription factors in cancer metastasis. International Journal of Molecular Sciences*,* 22 *(*7*),* 3591. DOI 10.3390/ijms22073591. 33808323 PMC8037257

[60] Shibue, T., Robert, A. W. (2017). EMT CSCs drug resistance. Nature Reviews Clinical Oncology*,* 14 *(*10*),* 611–629. DOI 10.1038/nrclinonc.2017.44. PMC5720366 28397828

[61] Saxena, M., Stephens, M. A., Pathak, H., Rangarajan, A. (2011). Transcription factors that mediate epithelial-mesenchymal transition lead to multidrug resistance by upregulating ABC transporters. Cell Death and Disease*,* 2 *(*7*),* e179–13. DOI 10.1038/cddis.2011.61. 21734725 PMC3199722

[62] Morel, A. P., Lièvre, M., Thomas, C., Hinkal, G., Ansieau, S. et al. (2008). Generation of breast cancer stem cells through epithelial-mesenchymal transition. PLoS One*,* 3 *(*8*),* 1–7. DOI 10.1371/journal.pone.0002888. PMC2492808 18682804

[63] Kurrey, N. K., Jalgaonkar, S. P., Joglekar, A. V., Ghanate, A. D., Chaskar, P. D. et al. (2009). Snail and slug mediate radioresistance and chemoresistance by antagonizing p53-mediated apoptosis and acquiring a stem-like phenotype in ovarian cancer cells. Stem Cells*,* 27 *(*9*),* 2059–2068. DOI 10.1002/stem.154. 19544473

[64] Lim, S., Becker, A., Zimmer, A., Lu, J., Buettner, R. et al. (2013). SNAI1-mediated epithelial-mesenchymal transition confers chemoresistance and cellular plasticity by regulating genes involved in cell death and stem cell maintenance. PLoS One*,* 8 *(*6*),* 1–12. DOI 10.1371/journal.pone.0066558. PMC3684605 23799116

[65] Vesuna, F., Lisok, A., Kimble, B., Raman, V. (2009). Twist modulates breast cancer stem cells by transcriptional regulation of CD24 expression. Neoplasia*,* 11 *(*12*),* 1318–1328. DOI 10.1593/neo.91084. 20019840 PMC2794513

[66] Lesniak, D., Sabri, S., Xu, Y., Graham, K., Bhatnagar, P. et al. (2013). Spontaneous epithelial-mesenchymal transition and resistance to HER-2-targeted therapies in HER-2-positive luminal breast cancer. PLoS One*,* 8 *(*8*),* 1–11. DOI 10.1371/journal.pone.0071987. PMC3753362 23991019

[67] Conn, S. J., Pillman, K. A., Toubia, J., Conn, V. M., Salmanidis, M. et al. (2015). The RNA binding protein quaking regulates formation of circRNAs. Cell*,* 160 *(*6*),* 1125–1134. DOI 10.1016/j.cell.2015.02.014. 25768908

[68] Chen, X., Chen, R. X., Wei, W. S., Li, Y. H., Feng, Z. H. et al. (2018). PRMT5 circular RNA promotes metastasis of urothelial carcinoma of the bladder through sponging miR-30c to induce epithelial-mesenchymal transition. Clinical Cancer Research*,* 24 *(*24*),* 6319–6330. DOI 10.1158/1078-0432.CCR-18-1270. 30305293

[69] Meng, J., Chen, S., Han, J. X., Qian, B., Wang, X. R. et al. (2018). Twist1 regulates vimentin through Cul2 circular RNA to promote EMT in hepatocellular carcinoma. Cancer Research*,* 78 *(*15*),* 4150–4162. DOI 10.1158/0008-5472.CAN-17-3009. 29844124

[70] Jian, X., He, H., Zhu, J., Zhang, Q., Zheng, Z. et al. (2020). Hsa_circ_001680 affects the proliferation and migration of CRC and mediates its chemoresistance by regulating BMI1 through miR-340. Molecular Cancer*,* 19 *(*1*),* 1–16. DOI 10.1186/s12943-020-1134-8. 32005118 PMC6993513

[71] Joseph, N. A., Chiou, S. H., Lung, Z., Yang, C. L., Lin, T. Y. et al. (2018). The role of HGF-MET pathway and CCDC66 cirRNA expression in EGFR resistance and epithelial-to-mesenchymal transition of lung adenocarcinoma cells. Journal of Hematology and Oncology*,* 11 *(*1*),* 1–14. DOI 10.1186/s13045-018-0557-9. 29855336 PMC5984410

[72] Hu, K., Liu, X., Li, Y., Li, Q., Xu, Y. et al. (2020). Exosomes mediated transfer of Circ-UBE2D2 enhances the resistance of breast cancer to tamoxifen by binding to MiR-200a-3p. Medical Science Monitor*,* 26*,* e922253. DOI 10.12659/MSM.922253. 32756532 PMC7431386

[73] Liu, Y. Y., Zhang, L. Y., Du, W. Z. (2019). Circular RNA circ-PVT1 contributes to paclitaxel resistance of gastric cancer cells through the regulation of ZEB1 expression by sponging miR-124-3p. Bioscience Reports*,* 39 *(*12*),* 1–11. DOI 10.1042/BSR20193045. PMC6928529 31793989

[74] Seimiya, T., Otsuka, M., Iwata, T., Shibata, C., Tanaka, E. et al. (2020). Emerging roles of exosomal circular RNAs in cancer. Frontiers in Cell and Developmental Biology*,* 8*,* 568366. DOI 10.3389/fcell.2020.568366. 33117799 PMC7578227

[75] Saber, S. H., Ali, H. E. A., Gaballa, R., Gaballah, M., Ali, H. I. et al. (2020). Exosomes are the driving force in preparing the soil for the metastatic seeds: Lessons from the prostate cancer. Cells*,* 9 *(*3*),* 564. DOI 10.3390/cells9030564. 32121073 PMC7140426

[76] Wei, H., Chen, Q., Lin, L., Sha, C., Li, T. et al. (2020). Regulation of exosome production and cargo sorting. International Journal of Biological Sciences*,* 17 *(*1*),* 163–177. DOI 10.7150/ijbs.53671. PMC7757038 33390841

[77] Ye, D., Gong, M., Deng, Y., Fang, S., Cao, Y. et al. (2022). Roles and clinical application of exosomal circRNAs in the diagnosis and treatment of malignant tumors. Journal of Translational Medicine*,* 20 *(*1*),* 1–17. DOI 10.1186/s12967-022-03367-x. 35382838 PMC8981684

[78] Kwok, Z. H., Wang, C., Jin, Y. (2021). Extracellular vesicle transportation and uptake by recipient cells: A critical process to regulate human diseases. Process*,* 9 *(*2*),* 273. DOI 10.3390/pr9020273. PMC8323758 34336602

[79] Turchinovich, A., Drapkina, O., Tonevitsky, A. (2019). Transcriptome of extracellular vesicles: State-of-the-art. Frontiers in Immunology*,* 10*,* 202. DOI 10.3389/fimmu.2019.00202. 30873152 PMC6404625

[80] Li, Y., Zheng, Q., Bao, C., Li, S., Guo, W. et al. (2015). Circular RNA is enriched and stable in exosomes: A promising biomarker for cancer diagnosis. Cell Research*,* 25 *(*8*),* 981–984. DOI 10.1038/cr.2015.82. 26138677 PMC4528056

[81] Kong, X., Li, G., Yuan, Y., He, Y., Wu, X. et al. (2012). MicroRNA-7 inhibits epithelial-to-mesenchymal transition and metastasis of breast cancer cells via targeting FAK expression. PLoS One*,* 7 *(*8*),* e41523. DOI 10.1371/journal.pone.0041523. 22876288 PMC3410899

[82] Tian, J. H., Liu, S. H., Yu, C. Y., Wu, L. G., Wang, L. B. (2021). The role of non-coding RNAs in Breast cancer drug resistance. Frontiers in Oncology*,* 11*,* 1–13. DOI 10.3389/fonc.2021.702082. PMC8473733 34589423

[83] Prihantono, F. M. (2021). Breast cancer resistance to chemotherapy: When should we suspect it and how can we prevent it? Annals of Medicine and Surgery*,* 70 *(*suppl 1*),* 102793. DOI 10.1016/j.amsu.2021.102793. 34691411 PMC8519754

[84] Ma, S., Kong, S., Wang, F., Ju, S. (2020). CircRNAs: Biogenesis, functions, and role in drug-resistant Tumours. Molecular Cancer*,* 19 *(*1*),* 1–19. DOI 10.1186/s12943-020-01231-4. 32758239 PMC7409473

[85] Liu, D., Fang, L. (2021). Current research on circular RNAs and their potential clinical implications in breast cancer. Cancer Biology and Medicine*,* 18 *(*3*),* 635–648. DOI 10.20892/j.issn.2095-3941.2020.0275. 34018386 PMC8330541

[86] Xu, T., Wang, M., Jiang, L., Ma, L., Wan, L. et al. (2020). CircRNAs in anticancer drug resistance: Recent advances and future potential. Molecular Cancer*,* 19 *(*1*),* 1–20. DOI 10.1186/s12943-020-01240-3. 32799866 PMC7429705

[87] Gong, L., Zhou, X., Sun, J. (2021). Circular rnas interaction with mirnas: Emerging roles in breast cancer. International Journal of Medical Sciences*,* 18 *(*14*),* 3182–3196. DOI 10.7150/ijms.62219. 34400888 PMC8364445

[88] Hua, X., Sun, Y., Chen, J., Wu, Y., Sha, J. et al. (2019). Circular RNAs in drug resistant tumors. Biomedicine and Pharmacotherapy*,* 118*,* 109233. DOI 10.1016/j.biopha.2019.109233. 31351436

[89] Wang, L., Yang, X., Zhou, F., Sun, X., Li, S. (2022). Circular RNA UBAP2 facilitates the cisplatin resistance of triple-negative breast cancer via microRNA-300/anti-silencing function 1B histone chaperone/PI3K/AKT/mTOR axis. Bioengineered*,* 13 *(*3*),* 7197–7208. DOI 10.1080/21655979.2022.2036894. 35263216 PMC8973968

[90] Yang, W., Yang, X., Wang, X., Gu, J., Zhou, D. et al. (2019). Silencing CDR1as enhances the sensitivity of breast cancer cells to drug resistance by acting as a miR-7 sponge to down-regulate REGγ. Journal of Cellular and Molecular Medicine*,* 23 *(*8*),* 4921–4932. DOI 10.1111/jcmm.14305. 31245927 PMC6652952

[91] Liu, D., Zhou, Z., Guo, Y., Du, Q., Li, L. (2022). CircCDK1 knockdown reduces CDK1 expression by targeting miR-489-3p to suppress the development of breast cancer and strengthen the sensitivity of Tamoxifen. Anticancer Drugs*,* 33 *(*3*),* 286–299. DOI 10.1097/CAD.0000000000001266. 34924499

[92] Sang, Y., Chen, B., Song, X., Li, Y., Liang, Y. et al. (2019). circRNA_0025202 regulates tamoxifen sensitivity and tumor progression via regulating the miR-182-5p/FOXO3a axis in breast cancer. Molecular Therapy*,* 27 *(*9*),* 1638–1652. DOI 10.1016/j.ymthe.2019.05.011. 31153828 PMC6731174

[93] Li, H., Li, Q., He, S. (2021). Hsa_circ_0025202 suppresses cell tumorigenesis and tamoxifen resistance via miR-197-3p/HIPK3 axis in breast cancer. World Journal of Surgical Oncology*,* 19 *(*1*),* 1–12. DOI 10.1186/s12957-021-02149-x. 33536026 PMC7860040

[94] Liang, X., Liu, X., Song, Z., Zhu, J., Zhang, J. (2022). Hsa_circ_0097922 promotes tamoxifen resistance and cell malignant behaviour of breast cancer cells by regulating ACTN4 expression via miR-876-3p. Clinical and Experimental Pharmacology and Physiology*,* 49 *(*12*),* 1257–1269. DOI 10.1111/1440-1681.13702. 35856314

[95] Liang, Y., Song, X., Li, Y., Ma, T., Su, P. et al. (2019). Targeting the circBMPR2/miR-553/USP4 axis as a potent therapeutic approach for breast cancer. Molecular Therapy-Nucleic Acids*,* 17*,* 347–361. DOI 10.1016/j.omtn.2019.05.005. 31302495 PMC6626870

[96] Zang, H., Li, Y., Zhang, X., Huang, G. (2020). Circ-RNF111 contributes to paclitaxel resistance in breast cancer by elevating E2F3 expression via miR-140-5p. Thoracic Cancer*,* 11 *(*7*),* 1891–1903. DOI 10.1111/1759-7714.13475. 32445273 PMC7327676

[97] Zheng, S. R., Huang, Q. Di, Zheng, Z. H., Zhang, Z. T., Guo, G. L. (2021). CircGFRA1 affects the sensitivity of triple-negative breast cancer cells to paclitaxel via the miR-361-5p/TLR4 pathway. Journal of Biochemistry*,* 169 *(*5*),* 601–611. DOI 10.1093/jb/mvaa148. 33481008

[98] Liu, G., Zhang, Z., Song, Q., Guo, Y., Bao, P. et al. (2020). Circ_0006528 contributes to paclitaxel resistance of breast cancer cells by regulating mir-1299/cdk8 axis. OncoTargets and Therapy*,* 13*,* 9497–9511. DOI 10.2147/OTT.S252886. 33061434 PMC7522311

[99] Ni, J., Xi, X., Xiao, S., Xiao, X. (2021). Silencing of circhipk3 sensitizes paclitaxel-resistant breast cancer cells to chemotherapy by regulating hk2 through targeting mir-1286. Cancer Management and Research*,* 13*,* 5573–5585. DOI 10.2147/CMAR.S307595. 34285578 PMC8285247

[100] Wang, L., Zhou, Y., Jiang, L., Lu, L., Dai, T. et al. (2021). CircWAC induces chemotherapeutic resistance in triple-negative breast cancer by targeting miR-142, upregulating WWP1 and activating the PI3K/AKT pathway. Molecular Cancer*,* 20 *(*1*),* 1–15. DOI 10.1186/s12943-021-01332-8. 33648498 PMC7919093

[101] Yang, W., Gu, J., Wang, X., Wang, Y., Feng, M. et al. (2019). Inhibition of circular RNA CDR1as increases chemosensitivity of 5-FU-resistant BC cells through up-regulating miR-7. Journal of Cellular and Molecular Medicine*,* 23 *(*5*),* 3166–3177. DOI 10.1111/jcmm.14171. 30884120 PMC6484300

[102] Zhu, M., Wang, Y., Wang, F., Li, L., Qiu, X. (2021). CircFBXL5 promotes the 5-FU resistance of breast cancer via modulating miR-216b/HMGA2 axis. Cancer Cell International*,* 21 *(*1*),* 1–12. DOI 10.1186/s12935-021-02088-3. 34281530 PMC8287742

[103] Wu, X., Ren, Y., Yao, R., Zhou, L., Fan, R. (2021). Circular RNA circ-MMP11 contributes to lapatinib resistance of breast cancer cells by regulating the miR-153-3p/ANLN axis. Frontiers in Oncology*,* 11*,* 1–15. DOI 10.3389/fonc.2021.639961. PMC8290203 34295807

[104] Yao, Y., Li, X., Cheng, L., Wu, X., Wu, B. (2021). Circular RNA FAT atypical cadherin 1 (circFAT1)/microRNA-525-5p/spindle and kinetochore-associated complex subunit 1 (SKA1) axis regulates oxaliplatin resistance in breast cancer by activating the notch and Wnt signaling pathway. Bioengineered*,* 12 *(*1*),* 4032–4043. DOI 10.1080/21655979.2021.1951929. 34288822 PMC8806415

[105] Liu, Y., Dong, Y., Zhao, L., Su, L., Luo, J. (2018). Circular RNA‐MTO1 suppresses breast cancer cell viability and reverses monastrol resistance through regulating the TRAF4/Eg5 axis. International Journal of Oncology*,* 53 *(*4*),* 1752–1762. DOI 10.3892/ijo.2018.4485. 30015883

[106] Wang, S., Wang, Y., Li, Q., Li, X., Feng, X. (2022). A novel circular RNA confers trastuzumab resistance in human epidermal growth factor receptor 2-positive breast cancer through regulating ferroptosis. Environmental Toxicology*,* 37 *(*7*),* 1597–1607. DOI 10.1002/tox.23509. 35234341

[107] Ling, Y., Liang, G., Lin, Q., Fang, X., Luo, Q. et al. (2022). circCDYL2 promotes trastuzumab resistance via sustaining HER2 downstream signaling in breast cancer. Molecular Cancer*,* 21 *(*1*),* 1–16. DOI 10.1186/s12943-021-01476-7. 34980129 PMC8722291

[108] Huang, L., Ma, J., Cui, M. (2021). Circular RNA hsa_circ_0001598 promotes programmed death-ligand-1-mediated immune escape and trastuzumab resistance via sponging miR-1184 in breast cancer cells. Journal of Immunology Research*,* 69 *(*6*),* 558–567. DOI 10.1007/s12026-021-09237-w. 34559381

[109] Huang, P., Li, F., Mo, Z., Geng, C., Wen, F. et al. (2021). A comprehensive RNA study to identify circRNA and miRNA biomarkers for docetaxel resistance in breast cancer. Frontiers in Oncology*,* 11*,* 1–14. DOI 10.3389/fonc.2021.669270. PMC8162208 34055636

[110] Liang, Y., Song, X., Li, Y., Su, P., Han, D. et al. (2019). circKDM4C suppresses tumor progression and attenuates doxorubicin resistance by regulating miR-548p/PBLD axis in breast cancer. Oncogene*,* 38 *(*42*),* 6850–6866. DOI 10.1038/s41388-019-0926-z. 31406252

[111] Wang, H., Shan, S., Wang, H., Wang, X. (2022). CircATXN7 contributes to the progression and doxorubicin resistance of breast cancer via modulating miR-149-5p/HOXA11 pathway. Anticancer Drugs*,* 33 *(*1*),* e700–e710. DOI 10.1097/CAD.0000000000001243. 34845164

[112] Chen, J., Shi, P., Zhang, J., Li, Y., Ma, J. et al. (2022). CircRNA_0044556 diminishes the sensitivity of triple-negative breast cancer cells to adriamycin by sponging miR-145 and regulating NRAS. Molecular Medicine Reports*,* 25 *(*2*),* 51. DOI 10.3892/mmr.2021.12567. 34913063 PMC8711030

[113] Dou, D., Ren, X., Han, M., Xu, X., Ge, X. et al. (2020). CircUBE2D2 (hsa_circ_0005728) promotes cell proliferation, metastasis and chemoresistance in triple-negative breast cancer by regulating miR-512-3p/CDCA3 axis. Cancer Cell International*,* 20 *(*1*),* 1–14. DOI 10.1186/s12935-020-01547-7. 32944002 PMC7491078

[114] Cui, Y., Fan, J., Shi, W., Zhou, Z. (2022). Circ_0001667 knockdown blocks cancer progression and attenuates adriamycin resistance by depleting NCOA3 via releasing miR-4458 in breast cancer. Drug Development Research*,* 83 *(*1*),* 75–87. DOI 10.1002/ddr.21845. 34227151

[115] Gao, D., Zhang, X., Beibei, L., Dong, M., Kai, F. et al. (2017). Screening circular RNA related to chemotherapeutic resistance in osteosarcoma by RNA sequencing. Epigenomics*,* 9 *(*9*),* 1175–1188. DOI 10.2217/epi-2017-0055. 28803498

[116] Mishra, A., Srivastava, A., Pateriya, A., Tomar, M. S., Mishra, A. K. et al. (2021). Metabolic reprograming confers tamoxifen resistance in breast cancer. Chemico-Biological Interactions*,* 347*,* 109602. DOI 10.1016/j.cbi.2021.109602. 34331906

[117] Mu, Q., Lv, Y., Luo, C., Liu, X., Huang, C. et al. (2021). Research progress on the functions and mechanism of circRNA in cisplatin resistance in tumors. Frontiers in Pharmacology*,* 12*,* 1–22. DOI 10.3389/fphar.2021.709324. PMC8458655 34566636

[118] Chen, X., Lu, P., Wu, Y., Wang, D. D., Zhou, S. et al. (2016). MiRNAs-mediated cisplatin resistance in breast cancer. Tumor Biology*,* 37 *(*10*),* 12905–12913. DOI 10.1007/s13277-016-5216-6. 27448297

[119] Ellis, L. M., Hicklin, D. J. (2009). Resistance to targeted therapies: Refining anticancer therapy in the era of molecular oncology. Clinical Cancer Research*,* 15 *(*24*),* 7471–7478. DOI 10.1158/1078-0432.CCR-09-1070. 20008847

[120] Lee, M., Koh, W. S., Han, S. S. (2003). Down-regulation of Raf-1 kinase is associated with paclitaxel resistance in human breast cancer MCF-7/Adr cells. Cancer Letters*,* 193 *(*1*),* 57–64. DOI 10.1016/S0304-3835(02)00722-X. 12691824

[121] Zhang, N., Yin, Y., Xu, S. J., Chen, W. S. (2008). 5-Fluorouracil: Mechanisms of resistance and reversal strategies. Molecules*,* 13 *(*8*),* 1551–1569. DOI 10.3390/molecules13081551. 18794772 PMC6244944

[122] Mader, R. M., Mu, M. (1998). Resistance to 5-Fluorouracil. General Pharmacology*,* 31 *(*5*),* 661–666. DOI 10.1016/S0306-3623(98)00191-8. 9809460

[123] Opdam, F. L., Guchelaar, H. J., Beijnen, J. H., Schellens, J. H. M. (2012). Lapatinib for advanced or metastatic breast cancer. Oncologist*,* 17 *(*4*),* 536–542. DOI 10.1634/theoncologist.2011-0461. 22477724 PMC3336826

[124] Garufi, C., Nisticò, C., Brienza, S., Vaccaro, A., D’Ottavio, A. et al. (2001). Single-agent oxaliplatin in pretreated advanced breast cancer patients: A phase II study. Annals of Oncology*,* 12 *(*2*),* 179–182. DOI 10.1023/A:1008386419047. 11300320

[125] Oshiro, C., Marsh, S., McLeod, H., Carrillo, M. W., Klein, T. et al. (2009). Taxane pathway. Pharmacogenet Genomics*,* 19 *(*12*),* 979–983. DOI 10.1097/FPC.0b013e3283335277. 21151855 PMC2998989

[126] Díaz, J. F., Andreu, J. M. (1993). Assembly of purified GDP-tubulin into microtubules induced by taxol and taxotere: Reversibility, ligand stoichiometry, and competition. Biochemistry*,* 32 *(*11*),* 2747–2755. DOI 10.1021/bi00062a003. 8096151

[127] Abal, M., Andreu, J., Barasoain, I. (2005). Taxanes: Microtubule and centrosome targets, and cell cycle dependent mechanisms of action. Current Cancer Drug Targets*,* 3 *(*3*),* 193–203. DOI 10.2174/1568009033481967. 12769688

[128] Nowak, A. K., Wilcken, N. R. C., Stockler, M. R., Hamilton, A., Ghersi, D. (2004). Systematic review of taxane-containing versus non-taxane-containing regimens for adjuvant and neoadjuvant treatment of early breast cancer. The Lancet Oncology*,* 5 *(*6*),* 372–380. DOI 10.1016/S1470-2045(04)01494-9. 15172358

[129] Cochran, J. C., Gatial, J. E., Kapoor, T. M., Gilbert, S. P. (2005). Monastrol inhibition of the mitotic kinesin Eg5. Journal of Biological Chemistry*,* 280 *(*13*),* 12658–12667. DOI 10.1074/jbc.M413140200. 15665380 PMC1356610

[130] Zhou, R., Wu, Y., Wang, W., Su, W., Liu, Y. et al. (2018). Circular RNAs (circRNAs) in cancer. Cancer Letters*,* 425 *(*1*),* 134–142. DOI 10.1016/j.canlet.2018.03.035. 29625140

[131] Salzman, J., Chen, R. E., Olsen, M. N., Wang, P. L., Brown, P. O. (2013). Cell-type specific features of circular RNA expression. PLoS Genetics*,* 9 *(*9*),* e1003777. DOI 10.1371/journal.pgen.1003777. 24039610 PMC3764148

[132] Afzal, S., Hassan, M., Ullah, S., Abbas, H., Tawakkal, F. et al. (2022). Breast cancer; Discovery of novel diagnostic biomarkers, drug resistance, and therapeutic implications. Frontiers in Molecular Biosciences*,* 9*,* 1–10. DOI 10.3389/fmolb.2022.783450. PMC8899313 35265667

[133] Jiang, Q., Liu, C., Li, C. P., Xu, S. S., Yao, M. D. et al. (2020). Circular RNA-ZNF532 regulates diabetes-induced retinal pericyte degeneration and vascular dysfunction. Journal of Clinical Investigation*,* 130 *(*7*),* 3833–3847. DOI 10.1172/JCI123353. 32343678 PMC7324174

[134] Zhang, Y., Nguyen, T. M., Zhang, X. O., Wang, L., Phan, T. et al. (2021). Optimized RNA-targeting CRISPR/Cas13d technology outperforms shRNA in identifying functional circRNAs. Genome Biology*,* 22 *(*1*),* 1–22. DOI 10.1186/s13059-021-02263-9. 33478577 PMC7818937

[135] He, A. T., Liu, J., Li, F., Yang, B. B. (2021). Targeting circular RNAs as a therapeutic approach: Current strategies andchallenges. Signal Transduction and Targeted Therapy*,* 6 *(*1*),* 185. DOI 10.1038/s41392-021-00569-5. 34016945 PMC8137869

[136] Yang, W., Du, W. W., Li, X., Yee, A. J., Yang, B. B. (2016). Foxo3 activity promoted by non-coding effects of circular RNA and Foxo3 pseudogene in the inhibition of tumor growth and angiogenesis. Oncogene*,* 35 *(*30*),* 3919–3931. DOI 10.1038/onc.2015.460. 26657152

[137] Han, D., Wang, Y., Wang, Y., Dai, X., Zhou, T. et al. (2020). The tumor-suppressive human circular RNA CircITCH sponges miR-330-5p to ameliorate doxorubicin-induced cardiotoxicity through upregulating SIRT6, survivin, and SERCA2a. Circulation Research*,* 127 *(*4*),* e108–e125. DOI 10.1161/CIRCRESAHA.119.316061. 32392088

[138] Liu, X., Abraham, J. M., Cheng, Y., Wang, Z., Wang, Z. et al. (2018). Synthetic circular RNA functions as a miR-21 sponge to suppress gastric carcinoma cell proliferation. Molecular Therapy. Nucleic Acids*,* 13*,* 312–321. DOI 10.1016/j.omtn.2018.09.010. 30326427 PMC6197335

[139] Du, W. W., Yang, W., Li, X., Awan, F. M., Yang, Z. et al. (2018). A circular RNA circ-DNMT1 enhances breast cancer progression by activating autophagy. Oncogene*,* 37 *(*44*),* 5829–5842. DOI 10.1038/s41388-018-0369-y. 29973691

[140] Kulkarni, J. A., Witzigmann, D., Chen, S., Cullis, P. R., van der Meel, R. (2019). Lipid nanoparticle technology for clinical translation of siRNA therapeutics. Accounts of Chemical Research*,* 52 *(*9*),* 2435–2444. DOI 10.1021/acs.accounts.9b00368. 31397996

[141] Wang, X., Zhang, H., Yang, H., Bai, M., Ning, T. et al. (2020). Exosome-delivered circRNA promotes glycolysis to induce chemoresistance through the miR-122-PKM2 axis in colorectal cancer. Molecular Oncology*,* 14 *(*3*),* 539–555. DOI 10.1002/1878-0261.12629. 31901148 PMC7053238

